# Supplementary material for: Adverse childhood experiences as a risk factor for depression-overweight comorbidity in adolescence and young adulthood
Source: Eur J Public Health. 2025 Jun 25;35(5):896–902. doi: 10.1093/eurpub/ckaf102 (PMC12529294; doi:10.1093/eurpub/ckaf102)
Supplement: ckaf102_Supplementary_Data [file ckaf102_supplementary_data.zip › ckaf102_Supplementary_Data/ejph-2024-08-om-0547-File003.docx]

**Supplementary File: Table S1.** Study definition of adverse childhood experiences (ACE), number of questionnaire items, informant, and number of retrospective items

|  | **Definition** | **Informant** | **Number of items** |
| --- | --- | --- | --- |
| Physical abuse | Partner/mother was physically cruel to child.  Adult in family pushed, grabbed, shoved/smacked to discipline child. People in child's family hit them so hard that it left them with bruises or marks. | Mother, partner | 36 |
| Sexual abuse | When growing up someone sexually abused child.  Touched in a sexual way by adult or older child, or was forced to touch adult or older child in a sexual way. Adult or older child forced, or attempted to force, child into any sexual activity by threatening or holding child down or hurting child in some way. | Mother, Child | 7 |
| Emotional abuse | Partner/mother emotionally cruel to child. Adult in family shouted/ said hurtful or insulting things. | Mother, partner | 37 |
| Emotional neglect | Carer knows who friends are.  Carer asks/starts conversation about free time/ what happened at school.  Carer takes time to listen when teenager talks about what happened in free time.  Discuss problems with anyone in their family.  Parent/carer talked about child’s experiences at school/ friends/ things that are troubling. Child feels left out of things.  Understood by parents.  When growing up there was someone to take respondent to the doctor if needed.  Someone in family made child feel important or special.  Carer knows what child does with other children. | Mother, Child | 19 |
| Bullying | Personal belongings stolen, threatened/blackmailed, hit/beaten up.  Do something didn't want to, told lies about child.  Friends tried to get teenager to do things didn’t want to / told lies about teenager.  Young person has been directly/relationally bullied. Child has been bullied.  Upset by name calling/exclusion from groups or bullying.  Someone threatened/blackmailed teenager. | Child | 17 |
| Parental substance abuse | Smoked cannabis.  Hard drug use or addiction (crack, heroin, amphetamine, opiate, cocaine, methadone, meth).  Alcoholism/drink problem. Score of >8 on the Alcohol Use Disorders Identification Test (AUDIT). | Mother, partner | 54 |
| Violence between parents | Physically cruel.  Kicked, bitten or hit each other, twisted arm, throw body, beaten each other up, choke or strangle each other.  Threatened or used knife or other weapon on each other. | Mother, partner | 46 |
| Parental criminal offence | Court conviction/convicted of an offense. | Mother, partner | 23 |
| Parental separation | Separated/divorced. | Mother, partner | 39 |
| Parental mental health problems or suicide attempt | Parent hurt themselves on purpose or attempted suicide.  Taken medication for depression or anxiety.  Score of >12 on the Edinburgh Postnatal Depression Scale (EPDS). Diagnosis of schizophrenia, bulimia, anorexia nervosa.  Hospital admission for psychiatric or mental health problems. | Mother, partner, child | 70 |
