# Supplementary material for: Adverse childhood experiences as a risk factor for depression-overweight comorbidity in adolescence and young adulthood
Source: Eur J Public Health. 2025 Jun 25;35(5):896–902. doi: 10.1093/eurpub/ckaf102 (PMC12529294; doi:10.1093/eurpub/ckaf102)
Supplement: ckaf102_Supplementary_Data [file ckaf102_supplementary_data.zip › ckaf102_Supplementary_Data/ejph-2024-08-om-0547-File004.docx]

**Supplementary File: Table S2.** Multiple imputation models of outcomes, exposures and covariates

| **Variable** | **Type** | **Missing data (%)** | **Model** | **Imputation model covariates** |
| --- | --- | --- | --- | --- |
| Sex | covariate | 0 | NA | NA |
| Ethnicity | covariate | 3 | logistic | ACE variables, education, class, financial difficulties, logged BMI at 17, depression at 17, logged BMI at 24, depression at 23, birthweight, gestational age, age of mother, parity, mother's smoking in pregnancy, housing tenure, parents' marital status |
| Physical abuse | exposure | 23 | logistic | ACE variables, social class, financial difficulties, logged BMI at 17, depression at 17, logged BMI at 24, depression at 23, ethnicity, birthweight, gestational age, age of mother, parity, mother's smoking in pregnancy, housing tenure, parents' marital status |
| Sexual abuse | exposure | 6 | logistic | ACE variables, social class, financial difficulties, logged BMI at 17, depression at 17, logged BMI at 24, depression at 23, ethnicity, birthweight, gestational age, age of mother, parity, mother's smoking in pregnancy, housing tenure, parents' marital status |
| Emotional abuse | exposure | 21 | logistic | ACE variables, social class, financial difficulties, logged BMI at 17, depression at 17, logged BMI at 24, depression at 23, ethnicity, birthweight, gestational age, age of mother, parity, mother's smoking in pregnancy, housing tenure, parents' marital status |
| Emotional neglect | exposure | 18 | logistic | ACE variables, social class, financial difficulties, logged BMI at 17, depression at 17, logged BMI at 24, depression at 23, ethnicity, birthweight, gestational age, age of mother, parity, mother's smoking in pregnancy, housing tenure, parents' marital status |
| Being bullied | exposure | 11 | logistic | ACE variables, social class, financial difficulties, logged BMI at 17, depression at 17, logged BMI at 24, depression at 23, ethnicity, birthweight, gestational age, age of mother, parity, mother's smoking in pregnancy, housing tenure, parents' marital status |
| Parental substance abuse | exposure | 19 | logistic | ACE variables, social class, financial difficulties, logged BMI at 17, depression at 17, logged BMI at 24, depression at 23, ethnicity, birthweight, gestational age, age of mother, parity, mother's smoking in pregnancy, housing tenure, parents' marital status |
| Violence between parents | exposure | 26 | logistic | ACE variables, social class, financial difficulties, logged BMI at 17, depression at 17, logged BMI at 24, depression at 23, ethnicity, birthweight, gestational age, age of mother, parity, mother's smoking in pregnancy, housing tenure, parents' marital status |
| Parental criminal offence | exposure | 17 | logistic | ACE variables, social class, financial difficulties, logged BMI at 17, depression at 17, logged BMI at 24, depression at 23, ethnicity, birthweight, gestational age, age of mother, parity, mother's smoking in pregnancy, housing tenure, parents' marital status |
| Parental separation | exposure | 23 | logistic | ACE variables, social class, financial difficulties, logged BMI at 17, depression at 17, logged BMI at 24, depression at 23, ethnicity, birthweight, gestational age, age of mother, parity, mother's smoking in pregnancy, housing tenure, parents' marital status |
| Parental mental health problems or suicide attempt | exposure | 17 | logistic | ACE variables, social class, financial difficulties, logged BMI at 17, depression at 17, logged BMI at 24, depression at 23, ethnicity, birthweight, gestational age, age of mother, parity, mother's smoking in pregnancy, housing tenure, parents' marital status |
| Parental education | covariate | 1 | multinomial logistic | ACE variables, social class, financial difficulties, logged BMI at 17, depression at 17, logged BMI at 24, depression at 23, ethnicity, birthweight, gestational age, age of mother, parity, mother's smoking in pregnancy, housing tenure, parents' marital status |
| Social class | covariate | 2 | multinomial logistic | ACE variables, education, financial difficulties, logged BMI at 17, depression at 17, logged BMI at 24, depression at 23, ethnicity, birthweight, gestational age, age of mother, parity, mother's smoking in pregnancy, housing tenure, parents' marital status |
| Financial difficulties | covariate | 5 | multinomial logistic | education, social class, logged BMI at 17, depression at 17, logged BMI at 24, depression at 23, ethnicity, maternal depressive score, maternal BMI, birthweight, gestational age, age of mother, parity, mother's smoking in pregnancy, housing tenure, parents' marital status |
| Maternal age | covariate | 1 | linear | education, social class, ethnicity |
| Logged BMI at age 17 | outcome | 4 | linear | ACE variables, overweight at age 15, depression at 17, depression at 23, education, class, financial difficulties, ethnicity, birthweight, gestational age, age of mother, parity, mother's smoking in pregnancy, housing tenure, parents' marital status |
| Depression at age 17 | outcome | 13 | logistic | ACE variables, depression at ages 16, 18, 21 and 22, logged BMI at 17, logged BMI at 24, education, class, financial difficulties, ethnicity, birthweight, gestational age, age of mother, parity, mother's smoking in pregnancy, housing tenure, parents' marital status |
| Logged BMI at age 24 | outcome | 39 | linear | ACE variables, overweight at age 15, depression at 17, depression at 23, education, class, financial difficulties, ethnicity, birthweight, gestational age, age of mother, parity, mother's smoking in pregnancy, housing tenure, parents' marital status |
| Depression at age 23 | outcome | 43 | logistic | ACE variables, depression at ages 16, 18, 21 and 22, logged BMI at 17, logged BMI at 24, education, class, financial difficulties, ethnicity, birthweight, gestational age, age of mother, parity, mother's smoking in pregnancy, housing tenure, parents' marital status |
