# Supplementary material for: Adverse childhood experiences as a risk factor for depression-overweight comorbidity in adolescence and young adulthood
Source: Eur J Public Health. 2025 Jun 25;35(5):896–902. doi: 10.1093/eurpub/ckaf102 (PMC12529294; doi:10.1093/eurpub/ckaf102)
Supplement: ckaf102_Supplementary_Data [file ckaf102_supplementary_data.zip › ckaf102_Supplementary_Data/ejph-2024-08-om-0547-File005.docx]

**Supplementary File: Table S3.** Unadjusted associations between adverse childhood experiences and depression-overweight comorbidity at age 17

|  | **Outcome** | | | | | | | | | |
| --- | --- | --- | --- | --- | --- | --- | --- | --- | --- | --- |
|  | **Ref: neither depression or overweight** | | | **Depression only** | | **Overweight only** | | | **Comorbidity** | |
| **Exposure** | **RRR** | **RRR** | **95% CI** | | **RRR** | | **95% CI** | **RRR** | | **95% CI** |
| **Ref: 0 ACEs** | 1 | 1 |  | | 1 | |  | 1 | |  |
| **1 ACE** |  | 1.44 | 1.07, 1.95 | | 1.33 | | 1.03, 1.72 | 1.37 | | 0.79, 2.37 |
| **2 to 3 ACEs** |  | 2.09 | 1.59, 2.76 | | 1.30 | | 1.02, 1.67 | 2.34 | | 1.45, 3.78 |
| **4 or more ACEs** |  | 3.72 | 2.74, 5.05 | | 1.33 | | 0.98, 1.81 | 3.65 | | 2.17, 6.15 |
| **Physical abuse** | 1 | 1.91 | 1.53, 2.39 | | 1.15 | | 0.91, 1.46 | 1.76 | | 1.19, 2.60 |
| **Sexual abuse** | 1 | 1.95 | 1.28, 2.96 | | 1.37 | | 0.87, 2.15 | 2.31 | | 1.34, 3.98 |
| **Emotional abuse** | 1 | 1.81 | 1.46, 2.24 | | 0.96 | | 0.76, 1.21 | 1.29 | | 0.86, 1.92 |
| **Emotional neglect** | 1 | 1.46 | 1.16, 1.86 | | 1.28 | | 1.03, 1.59 | 2.53 | | 1.78, 3.60 |
| **Being bullied** | 1 | 1.87 | 1.54, 2.27 | | 1.01 | | 0.83, 1.23 | 1.90 | | 1.39, 2.61 |
| **Parental substance abuse** | 1 | 1.51 | 1.12, 2.03 | | 1.01 | | 0.73, 1.40 | 1.13 | | 0.64, 1.98 |
| **Violence between parents** | 1 | 1.51 | 1.19, 1.91 | | 1.03 | | 0.81, 1.31 | 1.36 | | 0.92, 2.01 |
| **Parental criminal conviction** | 1 | 1.06 | 0.74, 1.53 | | 1.04 | | 0.75, 1.45 | 0.94 | | 0.50, 1.75 |
| **Parental separation** | 1 | 1.52 | 1.22, 1.89 | | 1.10 | | 0.89, 1.36 | 1.58 | | 1.11, 2.25 |
| **Parental mental health problems or suicide attempt** | 1 | 1.50 | 1.25, 1.81 | | 1.12 | | 0.94, 1.34 | 1.69 | | 1.25, 2.29 |

Note: Adjusted for sex. ACE=adverse childhood experiences, RRR=relative risk ratio, CI=confidence interval.
