# Supplementary material for: Adverse childhood experiences as a risk factor for depression-overweight comorbidity in adolescence and young adulthood
Source: Eur J Public Health. 2025 Jun 25;35(5):896–902. doi: 10.1093/eurpub/ckaf102 (PMC12529294; doi:10.1093/eurpub/ckaf102)
Supplement: ckaf102_Supplementary_Data [file ckaf102_supplementary_data.zip › ckaf102_Supplementary_Data/ejph-2024-08-om-0547-File006.docx]

**Supplementary File: Table S4.** Associations between adverse childhood experiences and depression-overweight comorbidity at age 17 in females

|  | **Outcome** | | | | | | | | | | | | | | | | | | |  |
| --- | --- | --- | --- | --- | --- | --- | --- | --- | --- | --- | --- | --- | --- | --- | --- | --- | --- | --- | --- | --- |
|  | **Ref: Neither depression or overweight** | | **Depression only** | | | | | | **Overweight only** | | | | | | **Comorbidity** | | | | |  |
|  |  | | **Unadjusted** | | **Adjusted** | | | | **Unadjusted** | | **Adjusted** | | | | **Unadjusted** | | **Adjusted** | | |  |
| **Exposure** | **RRR** | **RRR** | | **95% CI** | | **RRR** | **95% CI** | **RRR** | | **95% CI** | | **RRR** | **95% CI** | **RRR** | | **95% CI** | | **RRR** | **95% CI** | |
| **Ref: 0 ACEs** | 1 | 1 | |  | | 1 |  | 1 | |  | | 1 |  | 1 | |  | | 1 |  | |
| **1 ACE** |  | 1.60 | | 1.07, 2.41 | | 1.57 | 1.04, 2.37 | 1.74 | | 1.21, 2.49 | | 1.69 | 1.18, 2.42 | 1.49 | | 0.78, 2.84 | | 1.45 | 0.76, 2.76 | |
| **2 to 3 ACEs** |  | 2.45 | | 1.71, 3.52 | | 2.36 | 1.64, 3.39 | 1.65 | | 1.17, 2.33 | | 1.51 | 1.07, 2.14 | 2.58 | | 1.48, 4.49 | | 2.28 | 1.30, 3.99 | |
| **4 or more ACEs** |  | 4.78 | | 3.20, 7.15 | | 4.29 | 2.83, 6.51 | 1.65 | | 1.07, 2.55 | | 1.34 | 0.86, 2.11 | 4.58 | | 2.47, 8.50 | | 3.43 | 1.81, 6.49 | |
| **Physical abuse** | 1 | 2.13 | | 1.58, 2.86 | | 2.05 | 1.51, 2.79 | 1.12 | | 0.80, 1.56 | | 1.10 | 0.79, 1.54 | 1.96 | | 1.25, 3.07 | | 1.94 | 1.23, 3.06 | |
| **Sexual abuse** | 1 | 1.66 | | 1.06, 2.60 | | 1.53 | 0.97, 2.42 | 1.23 | | 0.74, 2.02 | | 1.12 | 0.68, 1.87 | 1.91 | | 1.05, 3.47 | | 1.61 | 0.87, 3.00 | |
| **Emotional abuse** | 1 | 2.11 | | 1.62, 2.76 | | 2.02 | 1.54, 2.65 | 0.98 | | 0.71, 1.36 | | 0.95 | 0.68, 1.31 | 1.36 | | 0.87, 2.12 | | 1.26 | 0.80, 2.00 | |
| **Emotional neglect** | 1 | 1.46 | | 1.08, 1.98 | | 1.37 | 1.01, 1.86 | 1.29 | | 0.95, 1.75 | | 1.17 | 0.86, 1.60 | 2.46 | | 1.61, 3.75 | | 2.07 | 1.35, 3.17 | |
| **Being bullied** | 1 | 1.88 | | 1.45, 2.45 | | 1.81 | 1.38, 2.36 | 0.94 | | 0.70, 1.27 | | 0.90 | 0.66, 1.22 | 1.97 | | 1.36, 2.86 | | 1.78 | 1.22, 2.61 | |
| **Parental substance abuse** | 1 | 1.52 | | 1.06, 2.19 | | 1.35 | 0.93, 1.96 | 0.81 | | 0.50, 1.31 | | 0.70 | 0.43, 1.15 | 0.96 | | 0.50, 1.86 | | 0.76 | 0.39, 1.48 | |
| **Violence between parents** | 1 | 1.57 | | 1.16, 2.12 | | 1.48 | 1.08, 2.01 | 0.97 | | 0.70, 1.35 | | 0.87 | 0.62, 1.22 | 1.30 | | 0.82, 2.06 | | 1.06 | 0.65, 1.73 | |
| **Parental criminal conviction** | 1 | 1.14 | | 0.72, 1.81 | | 1.11 | 0.69, 1.77 | 1.22 | | 0.78, 1.90 | | 1.21 | 0.77, 1.89 | 1.09 | | 0.54, 2.18 | | 1.08 | 0.53, 2.20 | |
| **Parental separation** | 1 | 1.67 | | 1.26, 2.22 | | 1.54 | 1.14, 2.07 | 1.36 | | 1.02, 1.79 | | 1.15 | 0.86, 1.54 | 2.03 | | 1.38, 2.99 | | 1.54 | 1.02, 2.34 | |
| **Parental mental health problems or suicide attempt** | 1 | 1.79 | | 1.40, 2.29 | | 1.69 | 1.32, 2.17 | 1.37 | | 1.07, 1.74 | | 1.29 | 1.01, 1.65 | 2.04 | | 1.42, 2.92 | | 1.85 | 1.29, 2.67 | |

Note: Adjusted for ethnicity, parental education, social class, financial difficulties and maternal age. ACE=adverse childhood experiences, RRR=relative risk ratio, CI=confidence interval.
