# Supplementary material for: Adverse childhood experiences as a risk factor for depression-overweight comorbidity in adolescence and young adulthood
Source: Eur J Public Health. 2025 Jun 25;35(5):896–902. doi: 10.1093/eurpub/ckaf102 (PMC12529294; doi:10.1093/eurpub/ckaf102)
Supplement: ckaf102_Supplementary_Data [file ckaf102_supplementary_data.zip › ckaf102_Supplementary_Data/ejph-2024-08-om-0547-File007.docx]

**Supplementary File: Table S5.** Associations between adverse childhood experiences and depression-overweight comorbidity at age 17 in males

|  | **Outcome** | | | | | | | | | | | | | | | | | | |  |
| --- | --- | --- | --- | --- | --- | --- | --- | --- | --- | --- | --- | --- | --- | --- | --- | --- | --- | --- | --- | --- |
|  | **Ref: Neither depression or overweight** | | **Depression only** | | | | | | **Overweight only** | | | | | | **Comorbidity** | | | | |  |
|  |  | | **Unadjusted** | | **Adjusted** | | | | **Unadjusted** | | **Adjusted** | | | | **Unadjusted** | | **Adjusted** | | |  |
| **Exposure** | **RRR** | **RRR** | | **95% CI** | | **RRR** | **95% CI** | **RRR** | | **95% CI** | | **RRR** | **95% CI** | **RRR** | | **95% CI** | | **RRR** | **95% CI** | |
| **Ref: 0 ACEs** | 1 | 1 | |  | | 1 |  | 1 | |  | | 1 |  | 1 | |  | | 1 |  | |
| **1 ACE** |  | 1.22 | | 0.75, 2.00 | | 1.19 | 0.73, 1.96 | 0.97 | | 0.68, 1.40 | | 0.96 | 0.67, 1.38 | 1.14 | | 0.37, 3.57 | | 1.12 | 0.36, 3.52 | |
| **2 to 3 ACEs** |  | 1.63 | | 1.02, 2.61 | | 1.56 | 0.97, 2.49 | 0.99 | | 0.69, 1.41 | | 0.96 | 0.66, 1.38 | 1.93 | | 0.73, 5.08 | | 1.81 | 0.68, 4.81 | |
| **4 or more ACEs** |  | 2.54 | | 1.57, 4.10 | | 2.31 | 1.42, 3.77 | 1.05 | | 0.67, 1.62 | | 1.02 | 0.65, 1.60 | 2.10 | | 0.71, 6.24 | | 2.00 | 0.66, 6.09 | |
| **Physical abuse** | 1 | 1.61 | | 1.10, 2.34 | | 1.56 | 1.06, 2.29 | 1.20 | | 0.85, 1.69 | | 1.21 | 0.85, 1.72 | 1.27 | | 0.53, 3.06 | | 1.26 | 0.52, 3.07 | |
| **Sexual abuse** | 1 | 4.94 | | 1.63, 14.92 | | 4.33 | 1.40, 13.41 | 2.54 | | 0.79, 8.22 | | 2.62 | 0.80, 8.58 | 9.03 | | 2.21, 36.95 | | 8.62 | 2.00, 37.10 | |
| **Emotional abuse** | 1 | 1.39 | | 0.98, 1.95 | | 1.36 | 0.96, 1.93 | 0.93 | | 0.65, 1.33 | | 0.96 | 0.67, 1.38 | 1.17 | | 0.52, 2.61 | | 1.22 | 0.54, 2.75 | |
| **Emotional neglect** | 1 | 1.46 | | 1.01, 2.13 | | 1.43 | 0.98, 2.08 | 1.27 | | 0.93, 1.74 | | 1.25 | 0.91, 1.73 | 2.73 | | 1.43, 5.24 | | 2.49 | 1.29, 4.81 | |
| **Being bullied** | 1 | 1.84 | | 1.36, 2.49 | | 1.90 | 1.40, 2.59 | 1.06 | | 0.81, 1.39 | | 1.08 | 0.82, 1.41 | 1.71 | | 0.93, 3.14 | | 1.81 | 0.97, 3.36 | |
| **Parental substance abuse** | 1 | 1.44 | | 0.88, 2.34 | | 1.34 | 0.82, 2.20 | 1.25 | | 0.80, 1.94 | | 1.21 | 0.77, 1.90 | 1.58 | | 0.57, 4.37 | | 1.52 | 0.54, 4.27 | |
| **Violence between parents** | 1 | 1.40 | | 0.97, 2.03 | | 1.31 | 0.90, 1.90 | 1.10 | | 0.77, 1.56 | | 1.06 | 0.74, 1.53 | 1.56 | | 0.74, 3.27 | | 1.51 | 0.71, 3.23 | |
| **Parental criminal conviction** | 1 | 0.96 | | 0.53, 1.75 | | 0.88 | 0.48, 1.61 | 0.85 | | 0.51, 1.44 | | 0.83 | 0.49, 1.40 | 0.57 | | 0.14, 2.35 | | 0.54 | 0.13, 2.28 | |
| **Parental separation** | 1 | 1.34 | | 0.94, 1.90 | | 1.21 | 0.84, 1.74 | 0.85 | | 0.61, 1.18 | | 0.76 | 0.54, 1.07 | 0.69 | | 0.30, 1.63 | | 0.59 | 0.24, 1.44 | |
| **Parental mental health problems or suicide attempt** | 1 | 1.16 | | 0.85, 1.57 | | 1.08 | 0.79, 1.47 | 0.89 | | 0.68, 1.17 | | 0.87 | 0.66, 1.15 | 1.08 | | 0.58, 1.98 | | 0.99 | 0.53, 1.85 | |

Note: Adjusted for ethnicity, parental education, social class, financial difficulties and maternal age. ACE=adverse childhood experiences, RRR=relative risk ratio, CI=confidence interval.
