# Supplementary material for: Adverse childhood experiences as a risk factor for depression-overweight comorbidity in adolescence and young adulthood
Source: Eur J Public Health. 2025 Jun 25;35(5):896–902. doi: 10.1093/eurpub/ckaf102 (PMC12529294; doi:10.1093/eurpub/ckaf102)
Supplement: ckaf102_Supplementary_Data [file ckaf102_supplementary_data.zip › ckaf102_Supplementary_Data/ejph-2024-08-om-0547-File008.docx]

**Supplementary File: Table S6.** Unadjusted associations between adverse childhood experiences and depression-overweight comorbidity at age 24

|  | **Outcome** |  |  | |  | |  |  | |  |
| --- | --- | --- | --- | --- | --- | --- | --- | --- | --- | --- |
|  | **Ref: neither depression or overweight** | | | **Depression only** | | **Overweight only** | | | **Comorbidity** | |
|  |  | | | **Unadjusted** | | **Unadjusted** | | | **Unadjusted** | |
|  | **RRR** | **RRR** | **95% CI** | | **RRR** | | **95% CI** | **RRR** | | **95% CI** |
| **Ref: 0 ACEs** | 1 | 1 |  | | 1 | |  | 1 | |  |
| **1 ACE** |  | 1.34 | 0.95, 1.90 | | 1.15 | | 0.91, 1.45 | 1.11 | | 0.75, 1.63 |
| **2 to 3 ACEs** |  | 1.81 | 1.28, 2.55 | | 1.19 | | 0.96, 1.48 | 1.83 | | 1.25, 2.67 |
| **4 or more ACEs** |  | 3.05 | 2.01, 4.63 | | 1.19 | | 0.89, 1.59 | 3.01 | | 1.93, 4.71 |
| **Physical abuse** | 1 | 1.97 | 1.47, 2.65 | | 0.98 | | 0.78, 1.23 | 1.93 | | 1.46, 2.56 |
| **Sexual abuse** | 1 | 2.34 | 1.41, 3.88 | | 1.47 | | 0.94, 2.29 | 3.56 | | 2.25, 5.65 |
| **Emotional abuse** | 1 | 1.61 | 1.19, 2.19 | | 1.02 | | 0.82, 1.26 | 1.52 | | 1.13, 2.04 |
| **Emotional neglect** | 1 | 1.07 | 0.80, 1.43 | | 1.07 | | 0.86, 1.34 | 1.43 | | 1.06, 1.94 |
| **Being bullied** | 1 | 1.54 | 1.17, 2.04 | | 1.01 | | 0.84, 1.21 | 1.56 | | 1.14, 2.12 |
| **Parental substance abuse** | 1 | 1.51 | 1.00, 2.29 | | 0.93 | | 0.67, 1.28 | 0.99 | | 0.61, 1.63 |
| **Violence between parents** | 1 | 1.54 | 1.10, 2.16 | | 1.21 | | 0.96, 1.53 | 1.62 | | 1.14, 2.32 |
| **Parental criminal conviction** | 1 | 1.32 | 0.85, 2.06 | | 0.88 | | 0.62, 1.24 | 1.32 | | 0.79, 2.21 |
| **Parental separation** | 1 | 1.43 | 1.07, 1.91 | | 1.15 | | 0.94, 1.41 | 1.56 | | 1.15, 2.12 |
| **Parental mental health problems or suicide attempt** | 1 | 1.51 | 1.18, 1.94 | | 1.10 | | 0.93, 1.30 | 1.74 | | 1.33, 2.28 |

Note: Adjusted for sex. ACE=adverse childhood experiences, RRR=relative risk ratio, CI=confidence interval.
