# Supplementary material for: Adverse childhood experiences as a risk factor for depression-overweight comorbidity in adolescence and young adulthood
Source: Eur J Public Health. 2025 Jun 25;35(5):896–902. doi: 10.1093/eurpub/ckaf102 (PMC12529294; doi:10.1093/eurpub/ckaf102)
Supplement: ckaf102_Supplementary_Data [file ckaf102_supplementary_data.zip › ckaf102_Supplementary_Data/ejph-2024-08-om-0547-File009.docx]

**Supplementary File: Table S7.** Associations between adverse childhood experiences and depression-overweight comorbidity at age 24 in females

|  | **Outcome** | | | | | | | | | | | | | | | | | | |  |
| --- | --- | --- | --- | --- | --- | --- | --- | --- | --- | --- | --- | --- | --- | --- | --- | --- | --- | --- | --- | --- |
|  | **Ref: Neither depression or overweight** | | **Depression only** | | | | | | **Overweight only** | | | | | | **Comorbidity** | | | | |  |
|  |  | | **Unadjusted** | | **Adjusted** | | | | **Unadjusted** | | **Adjusted** | | | | **Unadjusted** | | **Adjusted** | | |  |
| **Exposure** | **RRR** | **RRR** | | **95% CI** | | **RRR** | **95% CI** | **RRR** | | **95% CI** | | **RRR** | **95% CI** | **RRR** | | **95% CI** | | **RRR** | **95% CI** | |
| **Ref: 0 ACEs** | 1 | 1 | |  | | 1 |  | 1 | |  | | 1 |  | 1 | |  | | 1 |  | |
| **1 ACE** |  | 1.46 | | 0.96, 2.23 | | 1.46 | 0.95, 2.22 | 1.31 | | 0.95, 1.80 | | 1.28 | 0.92, 1.77 | 1.15 | | 0.71, 1.85 | | 1.11 | 0.69, 1.80 | |
| **2 to 3 ACEs** |  | 1.92 | | 1.26, 2.91 | | 1.92 | 1.26, 2.92 | 1.43 | | 1.05, 1.95 | | 1.31 | 0.96, 1.80 | 2.05 | | 1.34, 3.15 | | 1.85 | 1.20, 2.86 | |
| **4 or more ACEs** |  | 3.18 | | 1.93, 5.24 | | 3.10 | 1.87, 5.16 | 1.31 | | 0.90, 1.92 | | 1.06 | 0.71, 1.57 | 3.22 | | 1.92, 5.40 | | 2.46 | 1.42, 4.28 | |
| **Physical abuse** | 1 | 2.02 | | 1.43, 2.85 | | 2.01 | 1.41, 2.86 | 0.91 | | 0.66, 1.26 | | 0.89 | 0.64, 1.24 | 1.81 | | 1.28, 2.55 | | 1.79 | 1.26, 2.54 | |
| **Sexual abuse** | 1 | 1.70 | | 0.99, 2.91 | | 1.63 | 0.95, 2.81 | 1.54 | | 0.98, 2.42 | | 1.44 | 0.91, 2.29 | 2.75 | | 1.67, 4.51 | | 2.45 | 1.46, 4.11 | |
| **Emotional abuse** | 1 | 1.69 | | 1.17, 2.43 | | 1.66 | 1.15, 2.40 | 1.06 | | 0.79, 1.41 | | 1.02 | 0.76, 1.37 | 1.48 | | 1.03, 2.12 | | 1.41 | 0.97, 2.04 | |
| **Emotional neglect** | 1 | 1.37 | | 0.96, 1.95 | | 1.32 | 0.92, 1.91 | 1.09 | | 0.81, 1.48 | | 0.97 | 0.71, 1.32 | 1.87 | | 1.31, 2.67 | | 1.61 | 1.11, 2.32 | |
| **Being bullied** | 1 | 1.40 | | 0.99, 1.98 | | 1.37 | 0.97, 1.94 | 1.00 | | 0.76, 1.32 | | 0.94 | 0.71, 1.25 | 1.44 | | 0.99, 2.09 | | 1.31 | 0.89, 1.93 | |
| **Parental substance abuse** | 1 | 1.64 | | 1.01, 2.67 | | 1.57 | 0.95, 2.59 | 0.79 | | 0.51, 1.22 | | 0.68 | 0.43, 1.06 | 0.98 | | 0.57, 1.68 | | 0.77 | 0.44, 1.34 | |
| **Violence between parents** | 1 | 1.53 | | 1.04, 2.25 | | 1.52 | 1.03, 2.25 | 1.18 | | 0.88, 1.58 | | 1.08 | 0.80, 1.45 | 1.57 | | 1.02, 2.40 | | 1.35 | 0.86, 2.13 | |
| **Parental criminal conviction** | 1 | 1.11 | | 0.63, 1.96 | | 1.12 | 0.63, 1.98 | 0.89 | | 0.56, 1.42 | | 0.90 | 0.56, 1.43 | 1.31 | | 0.73, 2.35 | | 1.33 | 0.73, 2.41 | |
| **Parental separation** | 1 | 1.23 | | 0.87, 1.75 | | 1.16 | 0.81, 1.67 | 1.21 | | 0.92, 1.59 | | 1.01 | 0.76, 1.33 | 1.49 | | 1.04, 2.15 | | 1.14 | 0.77, 1.67 | |
| **Parental mental health problems or suicide attempt** | 1 | 1.66 | | 1.25, 2.22 | | 1.64 | 1.23, 2.20 | 1.29 | | 1.02, 1.62 | | 1.22 | 0.96, 1.55 | 2.11 | | 1.52, 2.93 | | 1.93 | 1.38, 2.69 | |

Note: Adjusted for ethnicity, parental education, social class, financial difficulties and maternal age. ACE=adverse childhood experiences, RRR=relative risk ratio, CI=confidence interval
