# Supplementary material for: Adverse childhood experiences as a risk factor for depression-overweight comorbidity in adolescence and young adulthood
Source: Eur J Public Health. 2025 Jun 25;35(5):896–902. doi: 10.1093/eurpub/ckaf102 (PMC12529294; doi:10.1093/eurpub/ckaf102)
Supplement: ckaf102_Supplementary_Data [file ckaf102_supplementary_data.zip › ckaf102_Supplementary_Data/ejph-2024-08-om-0547-File010.docx]

**Supplementary File: Table S8.** Associations between adverse childhood experiences and depression-overweight comorbidity at age 24 in males

|  | **Outcome** | | | | | | | | | | | | | | | | | | |
| --- | --- | --- | --- | --- | --- | --- | --- | --- | --- | --- | --- | --- | --- | --- | --- | --- | --- | --- | --- |
|  | **Ref: Neither depression or overweight** | | **Depression only** | | | | | | **Overweight only** | | | | | | **Comorbidity** | | | | |
|  |  | | **Unadjusted** | | **Adjusted** | | | | **Unadjusted** | | **Adjusted** | | | | **Unadjusted** | | **Adjusted** | | |
| **Exposure** | **RRR** | **RRR** | | **95% CI** | | **RRR** | **95% CI** | **RRR** | | **95% CI** | | **RRR** | **95% CI** | **RRR** | | **95% CI** | | **RRR** | **95% CI** |
| **Ref: 0 ACEs** | 1 | 1 | |  | | 1 |  | 1 | |  | | 1 |  | 1 | |  | | 1 |  |
| **1 ACE** |  | 1.18 | | 0.64, 2.17 | | 1.16 | 0.63, 2.13 | 0.99 | | 0.71, 1.37 | | 0.98 | 0.70, 1.36 | 1.05 | | 0.52, 2.12 | | 1.02 | 0.51, 2.07 |
| **2 to 3 ACEs** |  | 1.67 | | 0.94, 2.96 | | 1.61 | 0.90, 2.88 | 0.96 | | 0.70, 1.32 | | 0.94 | 0.68, 1.30 | 1.52 | | 0.74, 3.14 | | 1.43 | 0.68, 2.98 |
| **4 or more ACEs** |  | 2.86 | | 1.40, 5.82 | | 2.67 | 1.29, 5.51 | 1.06 | | 0.69, 1.65 | | 1.05 | 0.67, 1.63 | 2.71 | | 1.19, 6.17 | | 2.51 | 1.08, 5.85 |
| **Physical abuse** | 1 | 1.88 | | 1.11, 3.18 | | 1.85 | 1.08, 3.17 | 1.06 | | 0.74, 1.50 | | 1.09 | 0.76, 1.57 | 2.15 | | 1.26, 3.67 | | 2.21 | 1.26, 3.87 |
| **Sexual abuse** | 1 | 15.31 | | 2.33, 100.65 | | 16.51 | 2.39, 114.31 | 1.22 | | 0.13, 11.98 | | 1.23 | 0.12, 12.17 | 21.27 | | 3.35, 135.04 | | 22.63 | 3.38, 151.74 |
| **Emotional abuse** | 1 | 1.49 | | 0.90, 2.46 | | 1.46 | 0.88, 2.44 | 0.98 | | 0.70, 1.37 | | 1.03 | 0.73, 1.45 | 1.60 | | 0.92, 2.79 | | 1.68 | 0.94, 2.99 |
| **Emotional neglect** | 1 | 0.75 | | 0.44, 1.27 | | 0.72 | 0.42, 1.25 | 1.03 | | 0.76, 1.41 | | 1.02 | 0.75, 1.41 | 0.89 | | 0.52, 1.53 | | 0.86 | 0.50, 1.47 |
| **Being bullied** | 1 | 1.75 | | 1.16, 2.63 | | 1.86 | 1.22, 2.82 | 1.02 | | 0.79, 1.31 | | 1.02 | 0.79, 1.32 | 1.74 | | 1.03, 2.95 | | 1.82 | 1.06, 3.10 |
| **Parental substance abuse** | 1 | 1.28 | | 0.57, 2.84 | | 1.13 | 0.49, 2.59 | 1.07 | | 0.67, 1.72 | | 1.02 | 0.62, 1.66 | 0.97 | | 0.36, 2.61 | | 0.83 | 0.30, 2.28 |
| **Violence between parents** | 1 | 1.55 | | 0.86, 2.79 | | 1.42 | 0.78, 2.59 | 1.25 | | 0.89, 1.76 | | 1.21 | 0.86, 1.71 | 1.71 | | 0.91, 3.23 | | 1.55 | 0.81, 2.97 |
| **Parental criminal conviction** | 1 | 1.68 | | 0.82, 3.43 | | 1.61 | 0.78, 3.34 | 0.87 | | 0.53, 1.43 | | 0.85 | 0.51, 1.40 | 1.28 | | 0.49, 3.34 | | 1.20 | 0.45, 3.20 |
| **Parental separation** | 1 | 1.80 | | 1.09, 2.97 | | 1.61 | 0.97, 2.67 | 1.08 | | 0.79, 1.48 | | 0.98 | 0.71, 1.36 | 1.69 | | 0.98, 2.93 | | 1.39 | 0.79, 2.45 |
| **Parental mental health problems or suicide attempt** | 1 | 1.32 | | 0.85, 2.06 | | 1.27 | 0.81, 1.99 | 0.92 | | 0.71, 1.18 | | 0.90 | 0.70, 1.16 | 1.26 | | 0.77, 2.07 | | 1.17 | 0.71, 1.95 |

Note: Adjusted for ethnicity, parental education, social class, financial difficulties and maternal age. ACE=adverse childhood experiences, RRR=relative risk ratio, CI=confidence interval.
