# Supplementary material for: Adverse childhood experiences as a risk factor for depression-overweight comorbidity in adolescence and young adulthood
Source: Eur J Public Health. 2025 Jun 25;35(5):896–902. doi: 10.1093/eurpub/ckaf102 (PMC12529294; doi:10.1093/eurpub/ckaf102)
Supplement: ckaf102_Supplementary_Data [file ckaf102_supplementary_data.zip › ckaf102_Supplementary_Data/ejph-2024-08-om-0547-File011.docx]

**Supplementary File: Table S9.** Associations between adverse childhood experiences and depression-overweight comorbidity at age 17 in complete-case data

|  | **Outcome** | | | | | | | | | | | | | |
| --- | --- | --- | --- | --- | --- | --- | --- | --- | --- | --- | --- | --- | --- | --- |
|  | **Ref: neither depression or overweight** | **Depression only** | | | | **Overweight only** | | | | **Comorbidity** | | | |  |
|  |  | **Unadjusted** | | **Adjusted** | | **Unadjusted** | | **Adjusted** | | **Unadjusted** | | **Adjusted** | |  |
| **Exposure** | **RRR** | **RRR** | **95% CI** | **RRR** | **95% CI** | **RRR** | **95% CI** | **RRR** | **95% CI** | **RRR** | **95% CI** | **RRR** | **95% CI** | **P-value for sex interaction** |
| **Ref: 0 ACEs** | 1 | 1 |  | 1 |  | 1 |  | 1 |  | 1 |  | 1 |  | 0.4104 |
| **1 ACE** |  | 1.25 | 0.86, 1.81 | 1.21 | 0.83, 1.78 | 1.16 | 0.85, 1.58 | 1.13 | 0.82, 1.55 | 0.94 | 0.47, 1.88 | 0.84 | 0.41, 1.71 |  |
| **2 to 3 ACEs** |  | 1.91 | 1.35, 2.69 | 1.87 | 1.31, 2.67 | 1.14 | 0.84, 1.54 | 1.12 | 0.82, 1.53 | 2.03 | 1.12, 3.67 | 1.77 | 0.97, 3.23 |  |
| **4 or more ACEs** |  | 4.42 | 2.99, 6.53 | 4.05 | 2.69, 6.09 | 1.50 | 1.00, 2.23 | 1.45 | 0.96, 2.20 | 4.18 | 2.14, 8.16 | 3.50 | 1.75, 7.00 |  |
| **Physical abuse** | 1 | 2.07 | 1.63, 2.63 | 1.98 | 1.54, 2.53 | 1.22 | 0.94, 1.59 | 1.16 | 0.88, 1.52 | 2.05 | 1.37, 3.05 | 1.83 | 1.20, 2.81 | 0.3812 |
| **Sexual abuse** | 1 | 2.00 | 1.31, 3.04 | 1.93 | 1.24, 3.00 | 1.41 | 0.88, 2.28 | 1.36 | 0.82, 2.26 | 2.28 | 1.27, 4.10 | 1.83 | 0.95, 3.54 | 0.0774 |
| **Emotional abuse** | 1 | 1.87 | 1.49, 2.35 | 1.75 | 1.38, 2.22 | 0.99 | 0.76, 1.28 | 0.96 | 0.74, 1.26 | 1.32 | 0.87, 1.98 | 1.27 | 0.83, 1.94 | 0.2779 |
| **Emotional neglect** | 1 | 1.41 | 1.11, 1.79 | 1.39 | 1.09, 1.79 | 1.20 | 0.94, 1.52 | 1.06 | 0.82, 1.38 | 2.46 | 1.73, 3.52 | 2.07 | 1.42, 3.01 | 0.8198 |
| **Being bullied** | 1 | 1.90 | 1.55, 2.32 | 1.83 | 1.48, 2.25 | 1.10 | 0.89, 1.36 | 1.13 | 0.91, 1.40 | 2.01 | 1.44, 2.81 | 1.88 | 1.33, 2.67 | 0.6684 |
| **Parental substance abuse** | 1 | 1.51 | 1.10, 2.08 | 1.42 | 1.01, 1.98 | 1.01 | 0.70, 1.44 | 1.00 | 0.69, 1.44 | 1.22 | 0.69, 2.17 | 1.05 | 0.58, 1.91 | 0.2478 |
| **Violence between parents** | 1 | 1.52 | 1.18, 1.95 | 1.49 | 1.15, 1.94 | 0.98 | 0.75, 1.28 | 0.97 | 0.73, 1.28 | 1.46 | 0.95, 2.25 | 1.37 | 0.87, 2.15 | 0.6332 |
| **Parental criminal conviction** | 1 | 1.22 | 0.84, 1.76 | 1.23 | 0.84, 1.79 | 1.18 | 0.82, 1.71 | 1.25 | 0.86, 1.82 | 1.09 | 0.57, 2.06 | 1.02 | 0.52, 2.02 | 0.8129 |
| **Parental separation** | 1 | 1.54 | 1.23, 1.92 | 1.56 | 1.23, 1.97 | 1.08 | 0.86, 1.37 | 1.01 | 0.79, 1.30 | 1.57 | 1.08, 2.29 | 1.21 | 0.81, 1.81 | 0.1083 |
| **Parental mental health problems or suicide attempt** | 1 | 1.50 | 1.24, 1.82 | 1.37 | 1.12, 1.67 | 1.10 | 0.90, 1.33 | 1.04 | 0.86, 1.27 | 1.61 | 1.16, 2.23 | 1.48 | 1.06, 2.09 | 0.0096 |

Note: Adjusted for sex, ethnicity, parental education, social class, financial difficulties and maternal age. ACE=adverse childhood experiences, RRR=relative risk ratio, CI=confidence interval.

N ranged from 2340 (for adjusted association of ACEs categories) to 3718 (for unadjusted association of sexual abuse)
