# Supplementary material for: Adverse childhood experiences as a risk factor for depression-overweight comorbidity in adolescence and young adulthood
Source: Eur J Public Health. 2025 Jun 25;35(5):896–902. doi: 10.1093/eurpub/ckaf102 (PMC12529294; doi:10.1093/eurpub/ckaf102)
Supplement: ckaf102_Supplementary_Data [file ckaf102_supplementary_data.zip › ckaf102_Supplementary_Data/ejph-2024-08-om-0547-File012.docx]

**Supplementary File: Table S10.** Associations between adverse childhood experiences and depression-overweight comorbidity at age 24 in complete-case data

|  | **Outcome** | | | | | | | | | | | | | |
| --- | --- | --- | --- | --- | --- | --- | --- | --- | --- | --- | --- | --- | --- | --- |
|  | **Ref: neither depression or overweight** | **Depression only** | | | | **Overweight only** | | | | **Comorbidity** | | | |  |
|  |  | **Unadjusted** | | **Adjusted** | | **Unadjusted** | | **Adjusted** | | **Unadjusted** | | **Adjusted** | |  |
| **Exposure** | **RRR** | **RRR** | **95% CI** | **RRR** | **95% CI** | **RRR** | **95% CI** | **RRR** | **95% CI** | **RRR** | **95% CI** | **RRR** | **95% CI** | **P-value for sex interaction** |
| **Ref: 0 ACEs** | 1 | 1 |  | 1 |  | 1 |  | 1 |  | 1 |  | 1 |  | 0.4401 |
| **1 ACE** |  | 1.57 | 0.97, 2.54 | 1.63 | 0.98, 2.70 | 1.18 | 0.85, 1.63 | 1.13 | 0.81, 1.59 | 0.69 | 0.38, 1.26 | 0.60 | 0.32, 1.11 |  |
| **2 to 3 ACEs** |  | 1.92 | 1.21, 3.04 | 1.98 | 1.22, 3.23 | 1.37 | 1.00, 1.87 | 1.37 | 0.99, 1.89 | 1.67 | 1.02, 2.73 | 1.58 | 0.95, 2.62 |  |
| **4 or more ACEs** |  | 3.21 | 1.86, 5.56 | 3.32 | 1.86, 5.91 | 1.28 | 0.82, 1.98 | 1.23 | 0.78, 1.95 | 3.09 | 1.74, 5.51 | 2.76 | 1.52, 5.03 |  |
| **Physical abuse** | 1 | 2.02 | 1.46, 2.78 | 2.02 | 1.45, 2.83 | 1.12 | 0.85, 1.48 | 1.07 | 0.80, 1.42 | 2.46 | 1.73, 3.50 | 2.20 | 1.52, 3.19 | 0.0861 |
| **Sexual abuse** | 1 | 2.12 | 1.21, 3.73 | 2.06 | 1.13, 3.76 | 1.71 | 1.04, 2.82 | 1.71 | 1.01, 2.87 | 4.24 | 2.53, 7.12 | 4.00 | 2.30, 6.95 | 0.0835 |
| **Emotional abuse** | 1 | 1.74 | 1.25, 2.43 | 1.74 | 1.23, 2.46 | 1.07 | 0.81, 1.42 | 1.04 | 0.78, 1.39 | 1.77 | 1.22, 2.57 | 1.70 | 1.15, 2.51 | 0.1021 |
| **Emotional neglect** | 1 | 0.93 | 0.64, 1.36 | 0.98 | 0.66, 1.45 | 0.98 | 0.74, 1.29 | 0.94 | 0.70, 1.26 | 1.58 | 1.09, 2.29 | 1.50 | 1.02, 2.21 | 0.3835 |
| **Being bullied** | 1 | 1.46 | 1.08, 1.98 | 1.47 | 1.07, 2.03 | 0.91 | 0.71, 1.17 | 0.95 | 0.73, 1.23 | 1.53 | 1.08, 2.16 | 1.40 | 0.97, 2.01 | 0.5909 |
| **Parental substance abuse** | 1 | 1.80 | 1.10, 2.94 | 1.90 | 1.14, 3.17 | 1.15 | 0.75, 1.76 | 1.10 | 0.70, 1.73 | 0.82 | 0.40, 1.68 | 0.72 | 0.34, 1.52 | 0.1697 |
| **Violence between parents** | 1 | 1.62 | 1.12, 2.36 | 1.63 | 1.10, 2.41 | 1.33 | 0.99, 1.80 | 1.29 | 0.94, 1.77 | 1.81 | 1.20, 2.72 | 1.73 | 1.13, 2.65 | 0.8114 |
| **Parental criminal conviction** | 1 | 1.23 | 0.73, 2.08 | 1.24 | 0.72, 2.15 | 0.75 | 0.47, 1.18 | 0.82 | 0.52, 1.31 | 1.17 | 0.64, 2.13 | 1.18 | 0.63, 2.23 | 0.511 |
| **Parental separation** | 1 | 1.24 | 0.87, 1.75 | 1.19 | 0.83, 1.72 | 1.12 | 0.85, 1.47 | 0.97 | 0.73, 1.29 | 1.29 | 0.87, 1.91 | 0.99 | 0.65, 1.51 | 0.1683 |
| **Parental mental health problems or suicide attempt** | 1 | 1.49 | 1.12, 1.97 | 1.43 | 1.06, 1.93 | 1.15 | 0.92, 1.44 | 1.13 | 0.90, 1.42 | 1.96 | 1.42, 2.71 | 1.90 | 1.36, 2.66 | 0.1231 |

Note: Adjusted for sex, ethnicity, parental education, social class, financial difficulties and maternal age. ACE=adverse childhood experiences, RRR=relative risk ratio, CI=confidence interval.

N ranged from 1509 (for adjusted association of ACEs categories) to 2113 (for unadjusted association of sexual abuse
