# Supplementary material for: Adverse childhood experiences as a risk factor for depression-overweight comorbidity in adolescence and young adulthood
Source: Eur J Public Health. 2025 Jun 25;35(5):896–902. doi: 10.1093/eurpub/ckaf102 (PMC12529294; doi:10.1093/eurpub/ckaf102)
Supplement: ckaf102_Supplementary_Data [file ckaf102_supplementary_data.zip › ckaf102_Supplementary_Data/ejph-2024-08-om-0547-File013.docx]

**Supplementary File: Table S11.** The adjusted risk of having depression-overweigh comorbidity versus having neither depression or overweight associated with ACE score by level of parental education in complete-case data

|  |  | **Age 17 (n=2340)** | | **Age 24 (n=1509)** | |
| --- | --- | --- | --- | --- | --- |
|  |  | **RRR** | **95% CI** | **RRR** | **95% CI** |
| **Low parental education** | **Ref: 0 ACEs** | 1 |  | 1 |  |
|  | **1 ACE** | 0.74 | 0.30, 1.84 | 0.80 | 0.38, 1.69 |
|  | **2 to 3 ACEs** | 2.17 | 1.06, 4.42 | 2.01 | 1.07, 3.79 |
|  | **4 or more ACEs** | 4.19 | 1.88, 9.34 | 3.11 | 1.49, 6.51 |
|  |  |  |  |  |  |
| **High parental education** | **Ref: 0 ACEs** | 1 |  | 1 |  |
|  | **1 ACE** | 1.16 | 0.36, 3.80 | 0.34 | 0.10, 1.13 |
|  | **2 to 3 ACEs** | 1.17 | 0.37, 3.73 | 1.01 | 0.43, 2.40 |
|  | **4 or more ACEs** | 2.27 | 0.51, 10.11 | 2.56 | 0.88, 7.44 |
| **P-value for interaction with parental education** |  | 0.5576 |  | 0.7528 |  |

Note: Models are adjusted for sex, ethnicity, social class, financial difficulties and maternal age. ACE=adverse childhood experiences, RRR=relative risk ratio, CI=confidence interval.
