# Supplementary figures and images for: Adverse childhood experiences as a risk factor for depression-overweight comorbidity in adolescence and young adulthood
Source: Eur J Public Health. 2025 Jun 25;35(5):896–902. doi: 10.1093/eurpub/ckaf102 (PMC12529294; doi:10.1093/eurpub/ckaf102)

**Supplementary File: Figure S1.** Flow diagram of sample selection


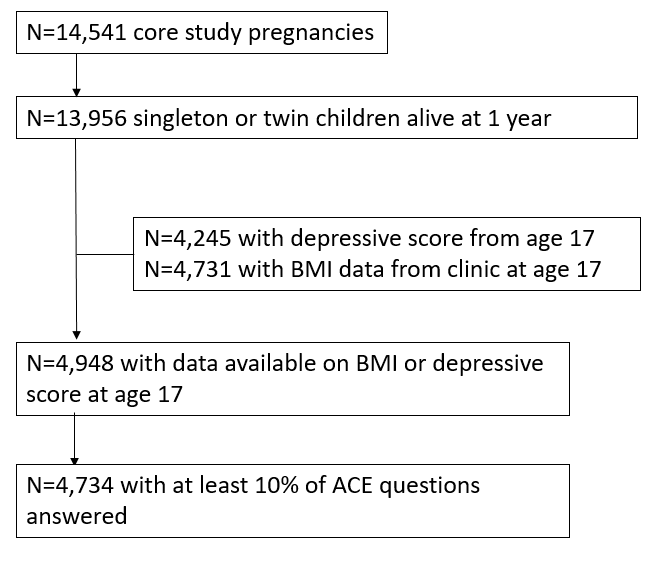

Supplement: ckaf102_Supplementary_Data [file ckaf102_supplementary_data.zip › ckaf102_Supplementary_Data/ejph-2024-08-om-0547-File002.docx]
